# The Effect of Physical Activity and Sedentary Behavior on Mental Health During the COVID-19 Pandemic Among the Adult Population in the Rural Area of Perambalur: A Cross-Sectional Study

**DOI:** 10.7759/cureus.36891

**Published:** 2023-03-29

**Authors:** Nazeemul K Azeem Jaffer, Tamilarasan Muniyapillai, Karthikeyan Kulothungan, Shagirunisha Rizvana, Sriranganathan Thirunavukkarasu

**Affiliations:** 1 Dermatology, Dhanalakshmi Srinivasan Medical College and Hospital, Perambalur, IND; 2 Community Medicine, Dhanalakshmi Srinivasan Medical College and Hospital, Perambalur, IND; 3 Community Medicine, Srinivasan Medical College and Hospital, Trichy, IND

**Keywords:** india, covid-19, sedentary behavior, mental health, physical activity

## Abstract

Background

The global COVID-19 pandemic has been incredibly destructive, especially for mental health. The lockdown measures required people to stay in their homes. This lifestyle caused them to become sedentary, which could have an impact on both their physical and mental well-being. We used the International Physical Activity Questionnaire (IPAQ) and the General Health Questionnaire-12 (GHQ-12) to measure the physical activity (PA) and mental health of adults in Perambalur, India, during the COVID-19 pandemic.

Materials and methods

The researchers conducted a cross-sectional investigation among people ages 15-60 years old from September 2021 to February 2022. In this study, we included 400 individuals through the method of convenient sampling. We conducted a population-based survey in which a semi-structured questionnaire was used to gather information on the age, gender, weight, height, physical activity (International Physical Activity Questionnaire {IPAQ}), and mental health (General Health Questionnaire-12 {GHQ-12}) of the participants. We conducted an analysis of the data using the Statistical Package for Social Sciences (SPSS) software version 20 (IBM SPSS Statistics, Armonk, NY).

Results

Most of the participants (65.8%) were female, and 69.5% belonged to the age group of 20-24 years; their mean age was 23 years. Physical activity was scored using the IPAQ, and we divided the participants into three categories: 37%, insufficient; 58%, sufficient; and 5%, high activity. The GHQ-12 assessment revealed that around half of the participants (47.8%) had psychological distress. In a bivariate analysis, those in the 15-19 and 24-29 age groups reported more distress than those in the other age groups (p = 0.006). Those who engaged in sufficient physical activity (54.7%) reported more distress than those who engaged in high (25%) or insufficient activity (p = 0.002).

Conclusion

Nearly half of the participants experienced psychological distress during the COVID-19 pandemic. Those who were engaging in sufficient physical activity experienced higher levels of distress than those with high and insufficient activities.

## Introduction

The Wuhan area of China experienced the introduction of a new coronavirus in December 2019. The virus spread rapidly and affected 45 people within a month [1]. By the end of January 2020, the state of Kerala had reported its first case of COVID-19. On March 10, 2020, there were 50 confirmed cases of COVID-19, and on March 12, 2020, the first fatality was announced. Afterward, COVID-19 spread rapidly throughout the country [2]. The second wave began in the middle of March 2021, and the peak number of cases (144,829) was recorded on April 9 [3]. In India, COVID-19 caused 3.2 million deaths between June 2020 and July 2021 of which 2.7 million occurred from April to July 2021 [4].

The pandemic measures caused significant changes in people's lifestyles. Previous studies noted the lack of physical activity (PA) and the deterioration in mental health [5]. The World Health Organization defines physical activity (PA) as "any physical movement produced by skeletal muscles that requires energy expenditure" [6]. It should engage in 75-150 minutes of severe aerobic exercise or a minimum of 150-300 minutes of moderate aerobic exercise every week. Multiple prior research articles have documented that regular and adequate amounts of physical activity have positive effects on the immune system and can help reduce the risk of obesity, diabetes, and psychological disorders [7].

There is an increasing trend of sedentary behavior and inactivity among the population, although it is difficult to determine whether COVID-19 control efforts contributed to this trend. Sedentary behavior refers to any awake behavior with an energy expenditure of less than 1.5 metabolic equivalents (METs) while in a sitting, reclining, or lying position [8]. There are two classifications for sedentary activities: nondiscretionary and discretionary. Nondiscretionary activities include sitting at work or school or when commuting by car or bus; discretionary activities include watching television, reading, using a computer, and playing video games [9].

In the late 1990s, researchers created the International Physical Activity Questionnaire (IPAQ) to encourage internationally comparable statistics on physical activity for health. They developed a short form of a questionnaire in order to survey the population's physical activity, and the information revealed the time spent sitting, walking, and carrying out moderate to strenuous physical activity [10].

Researchers have employed the General Health Questionnaire (GHQ), developed by Goldberg (1970), in a multitude of contexts and cultures for the psychological assessment of the general population [11]. The GHQ analyzes both positive and negative mental health states of an individual and can therefore assist healthcare practitioners in better understanding the psychological states of the public following the epidemic. This study examined the impact of physical activity on mental health during the COVID-19 pandemic. We used the International Physical Activity Questionnaire (IPAQ) and the General Health Questionnaire-12 (GHQ-12) to measure the health-related physical activity and mental health of adults in Perambalur during the COVID-19 pandemic.

## Materials and methods

Study design and setting

We conducted this population-based cross-sectional study among adults aged 15-60 years old in the rural area of Perambalur, Tamil Nadu, India, between the months of September 2021 and February 2022.

Ethical clearance and informed consent

Before the study began, we obtained an ethical clearance certificate from the Institutional Ethics Committee of Dhanalakshmi Srinivasan Medical College and Hospital (approval number: IECHS/IRCHS/N0: 120 B). Informed consent was obtained from all participants.

Selection criteria

We included people aged 15-60 and conducted in-person interviews to get data. We excluded all individuals with heart disease and kidney disease and those infected with COVID-19.

Sample size estimation and technique

According to a survey by Nair et al., 58.6% of people did little to no physical exercise during the pandemic [12]. We calculated the sample size using the formula n = 3.84 × P × Q / d^2^ (P = 58.6, Q = 41.4, and d = 5), and the sample size came up to 373. We collected responses from 400 members of the public in the urban and rural field practice areas of the teaching medical college in the district of Perambalur in Tamil Nadu using a convenience sample. The teaching medical college's field training area consists of 14 hamlets and 18 villages. In the urban and rural field practice areas of the tertiary care center in Perambalur, Tamil Nadu, there are about 8,690 rural and 9,010 urban households, according to the 2019 data from the rural and urban health centers.

Data collection procedure

A semi-structured questionnaire was used to collect data on the participant's age, gender, weight, and height; physical activity by International the Physical Activity Questionnaire (IPAQ) [13]; and mental health screening by General Health Questionnaire (GHQ) [11] through the interviewer method. At the time of the investigators' visit, they conducted interviews with all eligible individuals in the rural and urban field practice areas of a teaching medical college who met the inclusion criteria.

Measurement of General Health Questionnaire

The answer to every item was based on the Likert scale. We assigned the following code for positive questions: 3 = always, 2 = often, 1 = sometimes, and 0 = never. We then reversed the code for negative questions. We entered "often" and "always" as 1 and "sometimes" and "never" as 0. The researchers categorized the total score for mental health into GHQ ≥ 4 as psychological distress and GHQ < 4 as no psychological distress.

Measurement of Physical Activity by IPAQ

We used the IPAQ short-form questionnaire* *to determine the participants' habitual physical activity levels [13]. Theresults can be reported in categories (insufficient activity levels, sufficient activity levels, or high activity levels). Multiples of the estimated resting energy expenditure constitute a MET: (i) We considered the individuals to have insufficient physical activity* *if they did not meet the 600 MET minutes/week criteria for moderate or intense activity. (ii)* *To consider the individual to have* *sufficient activity, they must satisfy any of the following requirements: (a) three or more days of vigorous activity lasting at least 20 minutes each day, (b) five or more days of moderate activity lasting at least 30 minutes each day, and (c) five or more days of any combination of the aforementioned activities, with a combined weekly total of activity exceeding 600 MET minutes. (iii) To consider the individual to have high activity, they must satisfy any of the following criteria: (a) three or more days of intense activity totaling at least 1,500 MET minutes/week and (b) seven or more days of any combination of intense, moderate, or walking activity totaling at least 3,000 MET minutes/week.

A "seven-day recall" approach was used to collect the levels of physical activity. Those with a body mass index (BMI) lower than 18.5 kg/m^2^ can categorize as underweight, whereas those with a BMI above 27.5 kg/m^2^ classify as obese. We described the Asian-based classification of BMI for adults in Table 1 [14].

**Table 1:** Asian-based classification of body mass index (BMI) for adults Classification adopted from the study titled "Comparison of World Health Organization and Asia-Pacific body mass index classifications in COPD patients" and published in the International Journal of Chronic Obstructive Pulmonary Disease [14]

Body mass index (kg/m^2^)	Grade
Below 18.5	Underweight
18.5-22.9	Normal weight
23-27.5	Overweight
>27.5	Obese

Statistical analysis

We entered the collected data in Microsoft Excel (Microsoft® Corp., Redmond, WA) and analyzed the data using the Statistical Package for Social Sciences (SPSS) software 20 version (IBM SPSS Statistics, Armonk, NY). For descriptive statistics, we represented the continuous variable as mean and standard deviation (SD), whereas we represented categorical variables as frequencies and proportions. We used the chi-square test to determine the association between physical activity and sedentary behavior and mental health, and a p-value of <0.05 was statistically significant.

## Results

Most of the individuals in the study were female, making up 65.8%, and 69.5% were within the age range of 20-24 years. The mean age of the samples was 23.05, with a standard deviation of 5.36 years. Among the participants in the study, there were 69.5% with a normal BMI, 16% overweight, and 4.7% obese, as determined by the Asian criteria for BMI. We described the characteristics of the study participants in Table 2.

**Table 2:** Characteristics of study participants (n = 400) BMI: body mass index

Characteristics		Frequency (%)
Gender	Male	137 (34.2%)
	Female	263 (65.8%)
Age in years	15-19	79 (19.8%)
	20-24	278 (69.5%)
	25-29	22 (5.5%)
	>30	21 (5.2%)
BMI	Underweight	39 (9.8%)
	Normal	278 (69.5%)
	Overweight	64 (16%)
	Obese	19 (4.7%)

**Figure 1:**
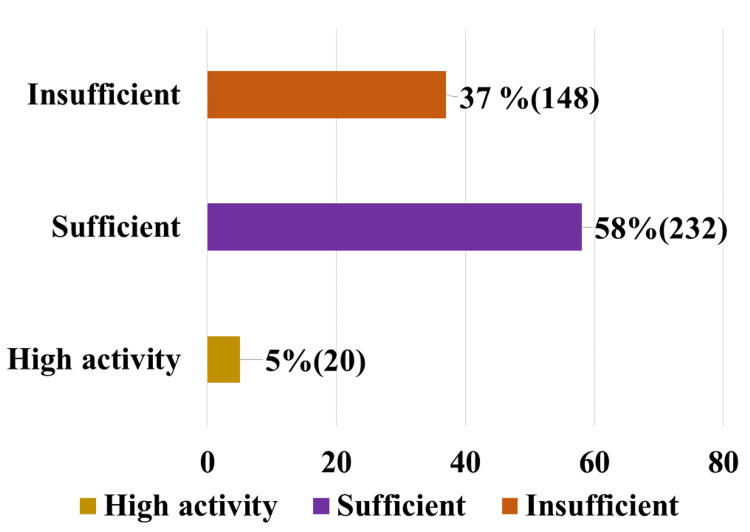
Assessment of physical activity based on IPAQ short form among study participants IPAQ: International Physical Activity Questionnaire

The IPAQ short form was used to examine physical activity levels, which were divided into three categories: insufficient, sufficient, and high activities, with 37%, 58%, and 5% of the sample population fitting into each of those categories, respectively. In Figure 1, we described the physical activity status based on the IPAQ short form among study participants.

**Figure 2:**
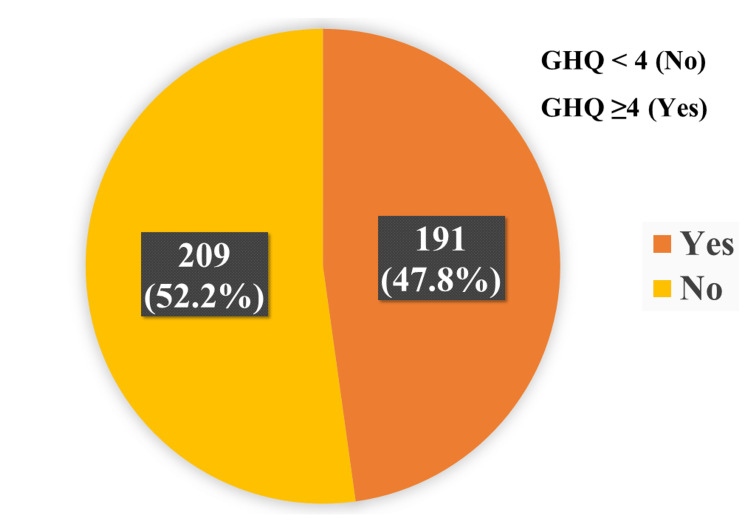
Assessment of mental health by using the General Health Questionnaire (GHQ)

We used the GHQ-12 as a screening tool to gauge the mental health of the study participants. The mean ± SD score for GHQ-12 was 3.67 ± 2.91, and nearly half of the participants (47.8%) experienced psychological distress during the COVID-19 pandemic. In Figure 2, we described the mental health status by using a general health questionnaire.

Researchers observed that 49% of females had psychological distress compared to males. The differences in psychological distress among different genders were not statistically significant (p = 0.471). The differences between the presence of psychological distress and BMI categories were not statistically significant (p = 0.792). Those in the 15-19 and 24-29 age groups experienced more distress than other age groups, and the difference was statistically significant (p = 0.006). Table 3 exhibited the association between the characteristics of the study participants and the mental health measure through the General Health Questionnaire.

**Table 3:** Association between the characteristics of study participants and mental health by GHQ-12 GHQ-12, General Health Questionnaire-12; BMI, body mass index

Characteristics		Psychological distress (GHQ-12)		P-value
		GHQ ≥ 4	GHQ <4	
Gender	Male	62 (45.3%)	75 (54.7%)	0.471
	Female	129 (49%)	134 (51%)	
Age	15-19	42 (53.2%)	37 (46.8%)	0.006
	20-24	132 (47.5%)	146 (52.55)	
	25-29	14 (63.6%)	8 (36.4%)	
	>30	3 (14.3%)	18 (85.7%)	
BMI	Underweight	18 (46.2%)	21 (53.8%)	0.792
	Normal	136 (48.9%)	142 (51.1%)	
	Overweight	30 (46.9%)	34 (53.1%)	
	Obese	7 (36.8%)	12 (63.2%)	

Those who were engaging in sufficient physical activity (54.7%) experienced higher levels of distress than those with high (25%) and insufficient (39.9%) activities, and this difference was statistically significant (p = 0.002). Table 4 presents the relationship between physical activity and mental health as measured by GHQ-12.

**Table 4:** Association between physical activity and mental health by GHQ-12 GHQ-12: General Health Questionnaire-12

Characteristics		Psychological distress (GHQ-12)		P-value
		GHQ ≥ 4	GHQ < 4	
Physical activity	High	5 (25%)	15 (75%)	0.002
	Sufficient	127 (54.7%)	105 (45.3%)	
	Insufficient	59 (39.9%)	89 (60.1%)	

## Discussion

We conducted this cross-sectional study among people ages 15-60 between September 2021 and February 2022. We excluded all individuals with heart disease and kidney disease and those infected with COVID-19. The "stay-at-home" orders issued by many governments throughout the world to stop the spread of COVID-19 altered people's routines [15].

The physical activity levels of the participants in the current study, as assessed by IPAQ, were 37% for insufficient physical activity, 58% for sufficient activity, and 5% for high activity. Ganesh et al. conducted a community-based online survey among 372 persons of both sexes aged 18-59 years in South India between November 2020 and March 2021, which revealed a decline in physical activity among their study participants [15]. Although the influence of pandemics on physical activities is unknown, the present study found that 37% of the study population had insufficient physical activity.

We determined that 47.8% of the sample population was experiencing some sort of psychological distress using the GHQ-12, a commonly used and validated screening instrument for mental health conditions. Sharma et al. conducted a meta-analysis of 21 online surveys conducted throughout the Indian subcontinent and published between 2020 and 2021. Only studies conducted with validated, standardized screening tools were included. Around 33% of the overall population reported experiencing psychological stress [16]. Higher levels of psychological distress were found in the current study, highlighting the need for further research.

In the event that COVID-19 is actively spreading across national borders, it is imperative that we address the needs of public health in order to resume normal community life. Public health initiatives and nondrug treatments have greater significance given that the majority of people have not received the COVID-19 vaccine. Promoting regular physical activity is crucial in the fight against the pandemic and for the prevention of health issues.

In our survey, it was determined that 4.7% of the 15-60 age group had obesity during the COVID-19 pandemic. A systematic review by de Rezende et al. in Brazil (2014) showed that there is firm evidence of a relationship between sedentary behavior and obesity [9]. In the present study, those who were engaging in sufficient physical activity experienced higher levels of distress than those with high and insufficient activities. Theis et al. undertook a study in the United Kingdom by 2021 that showed 61% reporting a reduction in physical activity levels and over 90% reporting a negative impact on mental health during the COVID-19 pandemic [17]. As this is a cross-sectional study about mental health and physical activity and nearly half of the participants in the study were in psychological distress, individuals who were stressed may have recently begun exercising owing to the abundance of free time during the pandemic and adopted a healthier lifestyle. The other possible explanation for this finding could be a spurious association, which is very common in noncommunicable diseases and their risk factors; i.e., the people at higher risk perform healthy lifestyle practices. Also, there is a possibility of social desirability bias, which could overestimate the physical activity and underestimate the psychological distress.

Limitation

We were unable to extrapolate the findings to other regions of the country because the study only included participants from a specific geographic area. Due to limited resources, the current study employed convenient sampling. The results of a probability sample may be more generalizable. The study used a method called "seven-day recall," which may not accurately show how active the population really is and may be prone to recall bias. The study made no note of other lifestyle components, such as dietary practices, sleeping hours, or alcohol or tobacco consumption. The temporal relationship between inactivity and mental health status cannot be determined, as this is a cross-sectional study. Further multicentric, probability sampling-based research is needed to completely comprehend the impact of physical inactivity on mental health during the COVID-19 pandemic.

## Conclusions

Nearly half of the participants experienced psychological distress during the COVID-19 pandemic. According to the International Physical Activity Questionnaire, nearly two-thirds of the individuals in the current study engage in sufficient physical activity, and only 5% were obese. Individuals who engaged in sufficient physical exercise reported higher levels of psychological distress, and this relationship requires additional research in different study settings.
